# Recent Trends of microRNA Significance in Pediatric Population Glioblastoma and Current Knowledge of Micro RNA Function in Glioblastoma Multiforme

**DOI:** 10.3390/ijms21093046

**Published:** 2020-04-27

**Authors:** Marek Mazurek, Cezary Grochowski, Jakub Litak, Ida Osuchowska, Ryszard Maciejewski, Piotr Kamieniak

**Affiliations:** 1 Department of Neurosurgery and Pediatric Neurosurgery, Medical University of Lublin, Jaczewskiego 8, 20-954 Lublin, Poland; marekmazurek@hotmail.com (M.M.); bea_43@o2.pl (P.K.); 2 Department of Anatomy, Medical University of Lublin, Jaczewskiego 4, 20-090 Lublin, Poland; i.m.osuchowska@gmail.com (I.O.); maciejewski.r@gmail.com (R.M.); 3 Department of Clinical Immunology, Medical University of Lublin, Jaczewskiego 8, 20-954 Lublin, Poland; jakub.litak@gmail.com

**Keywords:** glioblastoma multiforme, miRNA, RNA, glioma, high grade glioma, pediatric population, children

## Abstract

Central nervous system tumors are a significant problem for modern medicine because of their location. The explanation of the importance of microRNA (miRNA) in the development of cancerous changes plays an important role in this respect. The first papers describing the presence of miRNA were published in the 1990s. The role of miRNA has been pointed out in many medical conditions such as kidney disease, diabetes, neurodegenerative disorder, arthritis and cancer. There are several miRNAs responsible for invasiveness, apoptosis, resistance to treatment, angiogenesis, proliferation and immunology, and many others. The research conducted in recent years analyzing this group of tumors has shown the important role of miRNA in the course of gliomagenesis. These particles seem to participate in many stages of the development of cancer processes, such as proliferation, angiogenesis, regulation of apoptosis or cell resistance to cytostatics.

## 1. Introduction

Central nervous system tumors are a significant problem for modern medicine because of their location. It is estimated that about 250,000 patients receive inauspicious diagnoses each year [1]. In adults, approximately 32% of primary tumors show malignancy. Of these, glioblastoma multiforme (GBM) is the most common and also the most harmful diagnosis [2,3,4,5]. The standard treatment for this type of tumor is a combination of surgical resection, radiotherapy and chemotherapy [6,7]. Unfortunately, despite the implementation of appropriate therapy, attempts to improve patient prognoses and survival rates remain unsuccessful [3,4,6,8,9]. For this reason, many centers are conducting research in order to better understand the pathomechanisms of GBM development, which could allow us to find new treatments and improving existing ones. The explanation of the importance of microRNA (miRNA) in the development of cancerous changes plays an important role in this respect [10,11].

miRNAs are small, single-stranded RNA molecules with a length of 21 to 23 nucleotides [12,13,14]. They are encoded by the cell genome, as well as normal genes, while RNA polymerase II is responsible for their transcription [15,16]. The number of miRNAs encoded by the human genome is very divergent. These values range from about 600 to over 1900 (http://www.mirbase.org/cgi-bin/browse.pl?org=hsa [17,18]). It is estimated that they regulate around a third of all protein-coding genes and participate in such crucial processes as proliferation, cell differentiation and the mechanisms of apoptosis [19,20,21]. Interestingly, more than half of the genes encoding miRNAs are located in fragile chromosomal sites and other areas consistent with the development of cancerous processes [22,23]. The regulation mechanism relies on silencing the expression of some genes through binding in order to target messenger RNA (mRNA) [24]. Each miRNA is complementary, with a specific biological target, which is the corresponding mRNA fragment (usually located within the 3′ untranslated region). After fusion, the translation process is inhibited, preventing the expression of the encoded gene [25,26].

To this day, many authors have been able to demonstrate disturbances in the levels of various miRNAs in the course of GBM [26,27,28,29,30,31]. This applies to both the increased and decreased titers of miRNA compared to normal brain tissue [29,32,33,34,35,36,37,38,39,40,41,42,43,44,45] (Table 1). The significance of these differences has not yet been fully understood. In this study, we will try to introduce the role of miRNA in the pathogenesis of high-grade glioma among children.

## 2. Invasiveness

Tumor invasiveness is one of the aspects that is characteristic of GBM and is influenced by miRNA. It is conditioned by a number of features and mechanisms of the neoplasm process. These include epithelial to mesenchymal transition (EMT), which was first described in the 1980s in a paper about embryogenesis [46,47]. This involves changing the polarity of epithelial cells and their loss of ability to conduct cell–cell adhesion. As a result, their phenotype changes from epithelial to mesenchymal. This is manifested by a decrease in the expression of adhesive proteins such as E-cadherins, as well as the activation of new transcription factors, changes in the cytoskeleton of cells, the production of new enzymes capable of remodeling extracellular matrix components and the presentation of characteristic markers (*N*-cadherin, fibronectin, vimentin). The result is a decrease in the integrity of the environment and an increase in cell migration and tumor invasion [48,49,50]. In recent years, it has been discovered that miRNA can also influence the course of epithelial to mesenchymal transition. Studies on pancreatic cancer cell lines have shown that the presence of miR-655 causes EMT inhibition, resulting in decreased tumor cell migration and increased expression of E-cadherins. This process occurs through the effect on the TGF-β axis and the ZEB1 transcription factor, both of which normally promote epithelial to mesenchymal transition [51]. Similar relationships were observed for gliomas. They concerned, among others: miR-10-b, miR-21, miR-146 and miR-181b. The presence of some of them showed the effect of increasing tumor invasion (miR10-b, miR21), while others inhibited cell migration (miR-146) [52,53,54,55]. This also applies to high-grade gliomas, such as glioblastoma [56,57]. In studies conducted by Liao et al. on GBM cells, a decrease in miR-203 levels has been shown to be associated with increased EMT activity. The reason for this association was the effect of miR-203 on snail family transcriptional repressor 2 (SNAI2) [58]. It is an embryonic zinc finger protein whose function is to inhibit the expression of E-cadherins [59,60,61]. Liao et al. have shown that miR-203 causes the inhibition of SNAI2 expression, contributing to an increase in EMT activity and tumor invasion [58]. Furthermore, the researchers pointed out the connection between this relationship and the resistance of neoplasm cells to anticancer therapy, which is consistent with other papers describing SNAI2 [62,63]. The association of miRNA with the ZEB and SNAI2 axis was also suggested in other observations. Chang et al. indicated that tumor suppressor p53, through the activation of miR-200 may cause the inhibition of epithelial mesenchymal transition, affecting ZEB and SNAI2 production [64].

Matrix metalloproteinases (MMPs) are a group of proteins of special importance in the mechanisms of EMT and tumor invasion. These are endopeptidases involved in the regulation of cell metabolism and tissue remodeling processes [65,66]. Disorders of their function may cause the impairment of cell integrity and degradation of the extracellular matrix. This allows cells to disengage from the original environment and migrate, thereby increasing tumor invasiveness. The role of some of them (MMP-2, MMP-9, MMP-14) has been proved in the process of developing high grade gliomas [67,68,69,70,71]. In studies conducted by Gabriely et al., the effect of miR-21 on tumor aggressiveness was observed. Researchers have pointed to the ability of miR-21 to increase MMP activities by affecting the expression of a number of genes. The most important role was assigned to reversion-inducing-cysteine-rich protein with kazal motifs (RECK) and tissue inhibitor of metalloproteinases 3 (TIMP3). These are the genes responsible for the negative regulation of some MMPs. The inhibition of miR-21 has been shown to lead to an increase in RECK and TIMP3 levels, resulting in a decrease in MMP activities. This applied to both in vitro samples, as well as the human model of gliomas in mice. Interestingly, researchers suggested that only RECK is directly inhibited by miR-21 [53,72]. Other observations have shown that the effect of miR-21 on tumor invasion can also take place by inhibiting tumor suppressor genes ANP32A, SMARCA4 and sprouty homolog 2 (Spry2) [73,74]. The promotion of glioblastoma invasion by affecting metalloproteinases has also been demonstrated for miR-10b. The observations of Sun et al. have shown that these molecules affect HOXD10, which influences the expression of MMP14 and uPAR, contributing to greater tumor cell migration [75]. This fact is consistent with other studies reporting the correlation between the histological stage of gliomas and miR-10b expression [55].

On the contrary, in the study of Li et al. on glioblastoma cell lines, it was shown that miR-146b-5p has a negative effect on the activity of metalloproteinases (MMP2 and MMP16). The result was the inhibition of cell migration and tumor invasion [53]. Other researchers have also repeatedly pointed out the effect of miR-146 on the inhibition of tumor metastasis by suppressing MMPs [76,77,78]. The relationship between the presence of miRNA and the activity of metalloproteinases (MMP2, MMP3, MMP9, MMP12, MMP13, ADAM9) was demonstrated in many other observations conducted on gliomas, including glioblastoma [57,79,80,81,82,83,84,85].

Another example of miRNA associated with tumor invasion is miR-7. It has been shown that, in glioblastoma, its level is lowered compared to normal tissue and that it performs suppressor functions [44,86]. In addition, in this case, it was observed that it inhibits the activity of metalloproteinases (MMP2, MMP9) [26,56]. Moreover, it has an influence on metastasis by affecting certain genes such as RAF1, FAK, phosphatidylinositol 3-kinase (PI3K) and epidermal growth factor receptor (EGFR) [44,56,87,88]. Epidermal growth factor (EGF) is a protein, which, through binding to the receptor (EGFR) on the surface of cells, has a very large impact on their metabolism [89]. Phosphorylation of the transmembrane EGF receptor activates further signaling pathways, which include Akt, JNK and mitogen-activated protein kinase (MAPK) pathways, affecting cell proliferation and DNA synthesis, as well as other processes associated with tissue metabolism [90]. Receptor mutations often lead to its continuous activation, resulting in an uncontrolled increase in the number of cells [91]. In primary glioblastoma, EGFR is considered overexpressed in 63% of cases [92]. For GBM, the EGFRvIII-type mutation is characteristic [93,94,95,96]. In cancer cells, disorders in the EGFR are usually associated with an increase in cell migration, an increase in angiogenesis and an increase in tumor invasion [69,97,98]. A study performed by Kefas et al. confirmed that miR-7 levels are lowered in glioblastoma. Furthermore, they showed that miR-7 can inhibit the EGF receptor, thereby contributing to the inhibition of viability and the invasiveness of GMB cells. This process takes place both through the influence on the receptor itself as well as through insulin receptor substrates 1 and 2 (IRS-1 and IRS-2), which are regulators of the Akt pathway [44].

## 3. Apoptosis

Another characteristic feature of glioblastoma is the disorder of the mechanism of natural programmed cell death. Moreover, in this case, specific miRNA regulates tumor progression by inhibiting or increasing apoptosis [26]. The group of particles with antiapoptotic effects in the case of glioblastoma includes, among others, the previously described miR-21. Its effect on the mechanism of apoptosis includes many pathways, such as TGF-β, p53 and the mitochondrial apoptotic pathway [72].

**Figure 1:**
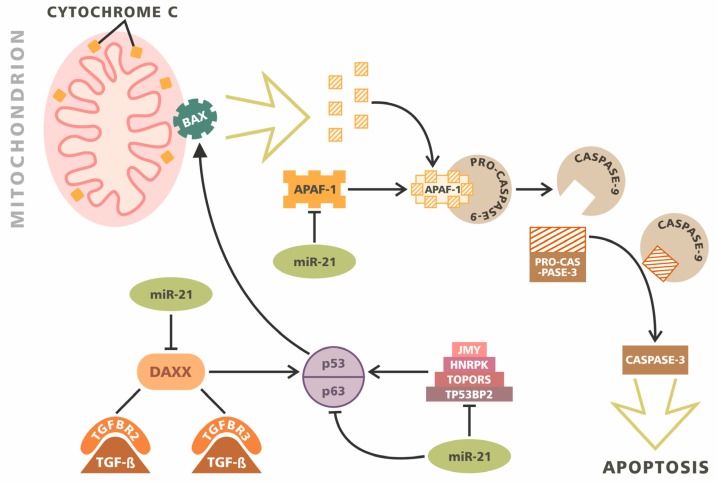
Multimodal inhibitory role of miR-21 in apoptosis. miR-21 directly inhibits the cascade of p53/p63 regulatory proteins, such as junction-mediating and regulatory protein (JMY), heterogeneous nuclear ribonucleoprotein K (HNRPK), TOPORS and tumor protein p53-binding protein 2 (TP53BP2). Additionally, miR-21 interacts with death-associated protein 6 (DAXX), the protein modulating TGF-beta/TGFBR2/3 signaling. The activation of BAX results in cytochrome C release. APAF-1 binds free cytochromes, creating APAF-1/PRO-CASPASE-9 complex. miR-21 inhibits APAF-1 expression and further activation of caspases. The activated CASPASE-9 cleaves PRO-CASPASE-3 to a mature form. CASPASE-3 triggers apoptosis. The miR-21 molecule indirectly limits CASPASE-3 activation and reduces apoptosis.

p53 is a tumor suppressor protein that plays key roles in the regulation of many aspects of cell metabolism, such as the cell cycle, genomic stability and apoptosis [99]. A similar role to p53 is played by its homologues: tumor protein p63 and tumor protein p73 [100]. The activation of p53 leads to the activation of numerous signal pathways containing (among others): junction-mediating and regulatory protein (JMY), death-associated protein 6 (DAXX), heterogeneous nuclear ribonucleoprotein K (HNRPK) and tumor protein p53-binding protein 2 (TP53BP2) [101,102,103,104]. Mutations in the p53 gene and disturbances in assisting proteins may be associated with abnormal cell division, the inhibition of their apoptosis and the impairment of internal repair mechanisms. So far, it has been possible to demonstrate their role in this type of disorder in the pathomechanism of most of the studied cancers [105]. This also applies to glioblastoma. Papagiannakopoulos et al. showed that the potential targets of miR-21 particles are p63 (p53 homolog), as well as HNRPK, TP53BP2, TOPORS, DAXX and JMY [72]. The overexpression miR-21 therefore leads to the disorder of these pathways, leading to pro-proliferative and antiapoptotic actions (Figure 1).

The TGF-β axis also plays an important role in the regulation of apoptosis in glioblastoma. It has been reported that miR-21 can regulate this pathway through the indirect impact of TGFB1/2 and the direct interaction of the receptors TGFBR2/3. Furthermore, it also affects the apoptotic mediator DAXX. Therefore, it constitutes a doubly important element, participating in the regulation of apoptosis both through the TGF-β axis and the p53 activation pathway. The effect of miR-21 on the mitochondrial apoptotic pathway is achieved through its interaction with APAF1 and PPIF [72].

Caspases are another group of enzymes involved in the regulation of programmed cell death. They exhibit cysteine protease activity, leading to apoptosis [106]. Studies by Chen et al. on glioblastoma cell lines have shown that reducing miR-21 levels can lead to an increase in caspase activity, resulting in the activation of the mechanism of apoptosis [34]. This was also confirmed in other studies pointing out the special role of caspase 3 and caspase 9 in this mechanism [107]. Furthermore, other miRNAs can also affect the activity of this group of enzymes. Austhakar et al. described the role of miR-211 in the activation of caspase 3 and caspase 9 in GBM [81]. Similar conclusions were made by Koueri et al. regarding miR-182 [108]. In addition, the significance of miRNA in the regulation of apoptosis conditioned by caspase activity in gliomas was also reported by other researchers [79,109,110]. The effect of miRNA-programmable cell death also takes place with the help of other mechanisms. It has been shown that miR-221 and miR-222 can affect the p53 pathway by interacting with the p53-upregulated modulator of apoptosis (PUMA) [26]. It is a protein belonging to the Bcl-2 family, and is capable of activating apoptosis mainly through the activation of p53 protein [111,112]. Research by Zhang et al. on glioblastoma cells showed that PUMA is the direct target of miR-221 and miR-222. An increase in the level of these miRNAs resulted in a decrease in PUMA activity and an increase in cell survival, while their reduced expression resulted in increased apoptosis [113]. Other studies also indicated the involvement of miR-221 and miR-222 in the regulation of programmable cell death in mechanisms independent of the p53 axis [114]. miR-335 is another molecule with antiapoptotic properties. Shu et al. described its effect on the disheveled-associated activator of morphogenesis 1 (DAAM1) in human malignant astrocytomas. DAAM1 is a potential tumor suppressor protein that inhibits the growth and invasiveness of the tumor. The inhibition of miR-335 leads to the induction of apoptosis, growth arrest and a decrease in tumor cell invasiveness in both in vivo and in vitro observations [115]. miR-17 has also been shown to have the ability to inhibit autophagy in GBM cells [116].

## 4. Angiogenesis

The mechanism of angiogenesis involves the formation of a network of new blood vessels based on existing ones [117]. It is particularly intensified in the case of high-grade tumors, including glioblastoma [118,119,120]. Studies carried out in recent years suggest that miRNA may also affect angiogenesis [121].

Numerous observations have shown that mechanisms of neovascularization can be both dependent and independent of the occurrence of hypoxia [69,122,123,124,125,126,127]. Researchers indicate that the main mechanism responsible for the formation of new blood vessels in glioblastoma is vascular endothelial growth factor (VEGF), which is directly related to hypoxia-inducible factor (HIF). HIFs are transcription factors that respond to fluctuations in the oxygen concentration in the cell environment [128,129,130].

**Figure 2:**
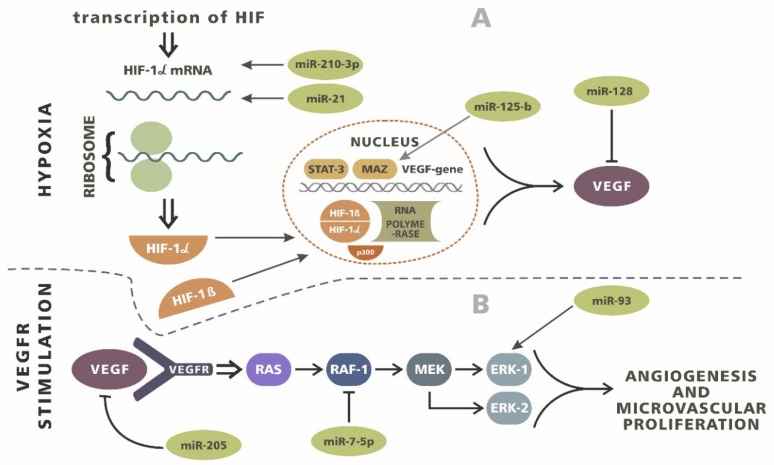
The influence of miRNA on angiogenesis process. (**A**) Hypoxia induces the transcription and Table 1. molecules. miR-210-3p and miR-21 act as a costimulators, intensifying expression. HIF-1 α cooperates with HIF-1β subunits and various complementary molecules such as p300, STAT-3 and myc-associated zinc finger protein (MAZ) during the transcription of vascular endothelial growth factor (VEGF). miR-125-b interplays with MAZ and a reduced level of miR-125-b results in an increase in MAZ and VEGF expression. miR-128 inhibits VEGF translation directly. (**B**) VEGF/VEGF receptor (R) axis signals through RAS/RAF-1/MEK/ERK-1/ERK-3. The promotion of this pathway results in intensive angiogenesis and microvascular proliferation. miR-205 inhibits VEGF/VEGFR interplay and miR 7-5p inhibits RAF-1. Both molecules significantly reduce angiogenesis in glioma. miR- 93 influences the expression of molecules by promoting microvascular growth.

A decrease in the level of HIF in response to hypoxia and the inactivation of prolyl hydroxylases cause the release of VEGF. The goal of this pathway is to counteract hypoxia by providing greater access to oxygen resources from the network of new vessels [69,125,131,132]. Studies performed by Hermansen et al. on patients with gliomas have shown that, within the tumor, increased levels of HIF-1α and VEGF are accompanied by an increase in miR-21 expression [133]. A similar mechanism was shown by Shi et al. regarding miR-128. However, in their observations, increased miRNA expression caused the opposite effect and a decrease in the severity of angiogenesis [134]. Wang et al. showed that, in the case of miR-143, this axis can be influenced by N-RAS [135]. The relationship between hypoxia-dependent angiogenesis and miRNA expression has also been proven in glioblastoma. Agrawal et al. showed that miR-210-3p expression correlates well with HIF-1α levels, as well as with other HIFs (HIF-3α). miR-210-3p expression increases the level of VEGF and the activity of carbonic anhydrase 9 (CA9), resulting in the development of the vascular network [136]. Wurdinger et al. suggested the effect of miR-296 on the induction of angiogenesis in GBM [137]. Moreover, Smith et al. showed a relationship between reduced miR-125-b expression in glioblastoma cells and an increase in myc-associated zinc finger protein (MAZ). MAZ is involved in the regulation of VEGF, which results in increased angiogenesis [138]. Numerous studies have reported the miRNA and VEGF interplay in glioma patients [82,139,140] (Figure 2).

VEGF is not the only mechanism affecting the severity of angiogenesis, which is affected by miRNAs. miR-93 is a member of the miR-17 family and its expression is increased in glioblastoma cells. Recent observations have shown that miR-93 is involved in the regulation of angiogenesis by affecting the expression of integrin-β8 [141]. It is a protein whose function is mediated by cell–cell and cell–extracellular matrix interactions [26,141]. Research by Tchaich et al. showed a relationship between the severity of angiogenesis and tumor invasion and the expression of integrin-β8. High levels of this protein were characteristic of lowly angiogenic and highly invasive neoplasms, while low expression meant a high capacity for vascular network development and low tumor invasiveness [142]. Observations made by Fang et al. on glioblastoma cells showed that integrin-β8 is one of the targets of miR-93 activity. The upregulation of miR-93 expression in the human glioblastoma cell line U87 resulted in an increase in vascularization [141]. An association with angiogenesis has also been suggested for other miRNAs belonging to the miR-17 family, such as miR-17-5p and miR-20a, as well as miR-29a, miR-155, miR-186, miR-429 and miR-675-5p [143,144,145,146,147,148].

One of the characteristic features of glioblastoma is also microvascular proliferation. This is useful for distinguishing gliomas with a lower degree of malignancy from GBM [75,87]. Liu et al. compared miR-7-5p levels in GBM microvessels and in healthy brain capillaries. Observations showed that miR-7-5p levels were significantly reduced for GBM. Moreover, an inverse correlation was observed between RAF1 and miR-7-5p levels due to the changes in the vascularization of the samples [87]. RAF1 is a serine/threonine kinase whose actions are based on the effect of mitogen-activated protein kinase (MAPK) activity, as it is a link in the Ras–Raf–MEK–ERK chain. Its role has already been observed in the pathomechanism of other cancers [149,150,151,152,153,154,155]. It has also been shown that increased RAF1 levels are present in human glioblastoma samples and that they may be associated with microvascular proliferation [156]. Moreover, Liu et al. have suggested that RAF1 is one of the potential targets of miR-7-5p. Increasing miR-7-5p levels in human umbilical vein endothelial cells resulted in a significant decrease in RAF1 expression [87].

## 5. Proliferation and Immunology

miRNAs also affect other processes that are important for the pathogenesis of tumors, such as cell cycle regulation and the control of cell proliferation. As previously mentioned, an example of this mechanism is miR-7 and its antiproliferative activity through the Akt axis [44,87]. Another particle with a similar effect is miR-128. It has been shown that, in glioblastoma, titers in the tissues of this miRNA usually remain low [157]. Its anti-proliferative effect is possible due to its influence on a number of metabolic pathways due to its effect on the expression of WEE1, EGFR, E2F3A and MSI1 [29,157,158,159]. E2F3A acts as a transcription factor and cell cycle activator through the retinoblastoma protein (RB1) and cyclin-dependent kinase (CDK)/cyclin complex genes. miR-124 and miR-137 also contribute to the regulation of this pathway and the cell cycle itself. They inhibit CDK6 activity and RB1 phosphorylation, thereby decreasing E2F pathway signaling [26]. miR-137 may also affect another gene involved in the regulation of proliferation, which is the previously mentioned MSI1 [160]. This is done through nuclear casein kinase and cyclin-dependent kinase substrate 1 (NUCKS1), which is a highly phosphorylated nuclear DNA-binding protein. It plays a role in recombination and DNA repair processes, and also acts as a substrate for cyclin-dependent kinase (CDK) [161,162,163,164,165]. The disorder of its expression has been associated with the pathogenesis of cancers, including brain tumors [166,167,168,169,170,171,172]. Its role in the regulation of proliferation has also been noted for many other miRNAs. This group includes, among others: miR-34a (via CDK6, CCND1 and NOTCH), miR6500-3a, let7 (via CCK-8), miR-21 (via PDCD4 and CASC2), miR-221 (via p27Kip1), miR-222 (via p27Kip1), miR-204 (via ATF2 and EZRIN), miR-23, miR-145 (via CTGF, NEDD9, NOTCH and platelet-derived growth factor receptors (PDGFR)), miR-181 (via NOTCH and SALL4), miR-29 (via QKI-6), miR-19a (via RUNX3), miR-101 (via SOX9), miR-107 (via NOTCH), miR-122 (via SOX2 and Wnt/β-catenin pathway), miR-140, miR-144, miR-152 (via RUNX2), miR-155 (via Wnt/β-catenin pathway), miR-182, miR-203 (via GAS41 and PCDH2), miR-186, miR-326, miR-384 [79,84,109,173,174,175,176,177,178,179,180,181,182,183,184,185,186,187,188,189,190,191,192,193,194,195,196,197,198,199,200,201,202,203,204].

miRNA also affects the regulation of the body’s immune processes. In terms of glioblastoma development, the programmed death ligand 1 (PD-L1) pathway is a particularly important factor [69,205,206,207,208]. The expression of PD-L1 on the surface of GBM cells causes the activation of the programmed death-1 (PD-1) receptor. As a result, the inhibition of T cell activity occurs, resulting in a decrease in the immune response and an increase in tumor invasiveness. This axis is regulated by many mechanisms, which also include miRNA expression [69]. A literature analysis carried out by Wang et al. pointed out the existence of 49 examples of miRNA participating in the regulation of the PD-1/PD-L1 axis. This concerned many cancers such as lung cancer, prostate cancer, kidney cancer, osteosarcoma, gastric cancer, melanoma, endometrial cancer, breast cancer and bladder cancer [209]. A similar mechanism, however, also occurs in gliomas. Research conducted by Wei et al. have shown that miR-138 can bind PD-1 by affecting the body’s immune response. Moreover, tests on animal models showed that miR-138 treatment may positively affect the results of treatment and reduce the value of tumor markers [210]. However, further research is needed to find out the exact characteristics of these processes for glioblastoma.

## 6. High Grade Gliomas and miRNA in the Pediatric Population

### 6.1. Expression of mRNA in Pediatric and Adult Population

Most studies analyzing the effect of miRNA on the pathogenesis of high-grade gliomas (HGGs) focus primarily on adult patients. To this day, relatively few observations have been made on the pediatric population. Studies that do refer to the pediatric population generally discuss such cancers as medulloblastoma, osteosarcoma, atypical teratoid/rhabdoid tumor (ATRT), leukemia and Ewing’s sarcoma [211,212,213,214,215,216,217,218,219,220]. Some researchers hae also focused their observations on brain tumors [221,222,223,224,225,226,227,228,229,230,231,232,233]. Pediatric HGGs constitute about 8–10% of all HGGs and, as in adults, these gliomas are characterized by high growth dynamics and a poor prognosis [234,235,236,237].

The first large-scale analysis of the occurrence of miRNA in the pediatric population was conducted by Birks et al. in 2011. They examined the differences between miRNA expression in pediatric brain tumors (ATRT, ependymoma, glioblastoma, medulloblastoma, and pilocytic astrocytoma) and normal tissue. The analysis was performed using the microarray method, taking into account 470 human miRNAs. In the case of pediatric GBM, the authors showed a significant reduction in miR-129 levels, as well as the overexpression of miR-25 and miR-142-5p [238]. These observations were consistent with previous work conducted on the adult patient population [31,33]. Distinctions in the occurrence of miR-129, miR-25 and miR-142-5p in the pediatric population have also been shown in a similar analysis by Miele et al. In this case, differences in miRNA levels between pediatric high-grade gliomas (pHGG), normal brain tissue and adult high-grade gliomas (aHGG) were studied. More miRNAs (754) were included in this, analysis of which 436 were expressed in HGG. Taking into account only the pediatric population, 152 miRNAs were found. The authors also showed that differential expression between pHGG and aHGG occurred in 228 cases. The most elevated miRNAs in pediatric samples compared to the adult population and normal brain tissue included: miR-15a, miR-17, miR-18a, miR-19a, miR-19b, miR-20, miR-27a, miR-92, miR-100, miR-106a, miR-195 and miR-497 (Table 2 and Table 3) [239].

An even more extensive analysis was carried out by Jha et al. They looked for expression of 1733 mature miRNA and 1658 pre-miRNAs in pediatric high-grade gliomas. Differences compared to the control samples were found in 266 cases. Overexpression concerned 55 mature miRNAs and two pre-miRNAs. The largest differences were found for miR-10b-5p, miR-891a-5p, miR-182-5p, miR-21-5p, miR-424-3p, miR-130b-3p, miR-130b-5p, miR-106b-3p and miR-4521. The opposite trend was shown in 71 mature miRNAs and 14 miRNA precursors, showing a reduced expression in the samples tested. This was most noticeable in the case of miR-7-5p, miR-137, miR-218-5p, miR-129-2-3p, miR-203a, miR-138-2-3p, miR-139-3p, miR-124-5p, miR-329-3p and miR-770-5p. In most cases, these results were consistent with the observations made in the adult population [240]. In the years that followed these studies, similar relationships were also studied by Giunti et al. In this case, differences in the expression of miR-137, miR-216a, miR-490, miR-501-3p, miR-521, miR-525-3p, miR-672, miR-873, miR-876-3p and miR-448 were demonstrated [172]. Another analysis was carried out by the team of Liang et al. In their work, they described the difference in miRNA expression by comparing pediatric low-grade gliomas (pLGG) with pediatric high-grade gliomas. They showed that significant overexpression in highly malignant tumors concerned only miR-17-5p and miR-561-5p, whereas a reduced level was detected for miR-137 and miR-6500-3p [174] (Table 4).

### 6.2. Tumorgenesis—Similarities and Differences

The differences presented in the above studies are associated with the different courses of tumorgenesis in adult and pediatric glioblastomas. For the adult population, there are two GBM subtypes:

- **Primary**—these are tumors that develop rapidly de novo;
- **Secondary**—these are derived from the evolution of low-grade gliomas that acquire malignancy.

The different starting point of tumorgenesis also causes discrepancies in genetic profiling and the molecular pathways of tumor metabolism. Primary GBMs are often characterized by mutations in the phosphatase and tensin homolog deleted on chromosome ten (PTEN) range and EGFR amplification, while, in secondary glioblastoma, we are more often dealing with TP53 mutations [8,176,237,238,239,241,242,243,244,245,246,247,248]. An analysis of the activity of pediatric GBM signaling pathways shows that, in this manner, they are similar to the secondary subtype [172]. This also applies to PTEN status and the associated phosphatidylinositol 3-kinase (PI3K) pathway. The activation of PI3K results in the formation of phosphatidylinositol-3,4,5,-triphosphate (PIP3), which results in the phosphorylation of Akt and its location in the plasma membrane. This transformation can lead to the activation of the kinase function, leading to uncontrolled cell proliferation and the inhibition of apoptosis as a result of tumor transformation [248,249,250,251]. The PTEN gene, which is a suppressor gene and a natural PI3K/AKT axis inhibitor, plays an important role in regulating this pathway [252,253]. It encodes an enzyme with phosphatase activity, which is capable of dephosphorylating PIP3, leading to the inhibition of Akt activity. Mutations in the PTEN gene leading to its abnormal function, resulting in the continuous activity of Akt and leading to the tumorogenesis process [254]. This phenomenon has been observed in gliomas, including multiform glioblastomas [255]. Moreover, PTEN mutations are relatively common in the adult population (they occur in about 50% of cases), while they are rare among children [237,256,257,258,259]. Observations made in recent years have shown that the regulation of the expression of this gene may also be affected by miRNAs. Studies of the adult population have revealed that the miRNAs that may affect regulation include miR-21, miR-26, miR-221, miR-222 [260,261,262]. Observations made by Miele et al. show that a similar phenomenon may also apply to the pediatric population. In their work, the authors suggested that the overexpression of the miR-17-92 cluster may cause the quenching of PTEN gene activity. The effect of this is an increase in cell proliferation and the acceleration of the tumor processes [239]. The miR-17-92 cluster is a larger group of miRNAs comprising six families, which include miR-17 (miR-17, miR-20a), miR-18 (miR-18a), miR-19 (miR-19a, miR-19b), and miR-92 (miR-92a) [263]. Its overexpression in the pediatric population was also noted in a paper by Jha et al. [240]. The attention of Miele et al. was also drawn to the effect of the same miRNA group on the expression of the retinoblastoma protein (RB1) gene [239]. It is a suppressor gene responsible for the regulation of the cell cycle [264]. RB1 mutations occur in glioblastomas in children, but they are less common than in the adult population [258,259]. Miele et al., in their work, showed that cluster miR-17-92 can silence the action of the RB1 gene, contributing to the intensification of gliomagenesis [239]. It is worth noting that the authors also emphasized the impact of this group of miRNAs on the Sonic Hedgehog pathway involved in the transmission of information on embryonic cell differentiation [239,265]. Many authors have pointed out the role of the Sonic Hedgehog pathway in controlling cell proliferation via the miR-17-92 cluster in both physiological brain development and tumor pathogenesis [214,266,267,268]. This also applies to gliomas in adults [266,267]. Miele et al. showed the disorders of this pathway in the pediatric population [239].

### 6.3. The Role of Growth Factors

Growth factor receptors are other elements of the PI3K axis that are involved in its activation. These include EGFR, EGFRvIII and platelet-derived growth factor receptors (PDGFR). They are also factors that can differentiate between both primary and secondary glioblastoma, as well as between aGBM and pGMB. The occurrence of EGFR overexpression in the pediatric population, which is characteristic of the primary subtype, is rarer (compared to the adult population) and the upregulation of PDGFR is more frequent [269,270,271,272]. As mentioned earlier, EGFR is a receptor protein located in the cell membrane that is involved in the activation of a number of signaling pathways. It is estimated that overexpression occurs in 30–50% of glioblastomas in the adult population, but overexpression only occurs in about 3% of glioblastomas in children [237,241,273]. PDGFR is involved in the regulation of cell proliferation and differentiation, as well as angiogenesis [274,275]. Both types of receptors play important functions in the metabolism of cells, but studies on a mouse model suggest that PDGF is the most important in the induction of gliomagenesis [276,277,278,279]. Interestingly, the work of Yang et al. suggests that miRNA may also be involved in the regulation of these signaling pathways. In their experiment, conducted on glioblastoma cells and glioma stem cell (GSC) lines, the authors demonstrated the ability of miR-29a to affect PDGF expression, thereby inhibiting growth and the ability of cancer cells to migrate, inducing their apoptosis [280]. Moreover, the observations of Costa et al. in a study performed on glioblastoma cells have shown that PDGF alone can affect the expression of some pro- and anti-oncogenic miRNAs. They showed the inhibitory effect of PDGF-B on miR-21 and miR-128 [281]. This demonstrates the complexity of the glioblastoma pathomechanism and emphasizes the need for further research into this matter. Data regarding the significance of the PDGF axis in gliomagenesis have also been confirmed by a large analysis of genomic imbalances in childhood high-grade gliomas by Pough et al. In their studies, PDGFR expression was high, while EGFR was significantly underexpressed. Moreover, the authors noted the lack of IDH1 hotspot mutation [241]. IDH status is another feature of the glioblastoma subtype, the expression of which varies between age groups. The IDH gene, with its two isoforms (IDH1 and IDH2), encodes isocitrate dehydrogenase, which is the enzyme responsible for the oxidative decarboxylation of isocitrate. Its mutation causes the transformation of α-ketoglutarate to (D)-2-hydroxyglutarate, which results in the hypermethylation of DNA and histones, which may contribute to tumor development [282,283,284]. These types of changes are rare in pGBS (<10%), while their occurrence in the adult population is very common (98%) [241,272,285,286,287,288,289,290,291,292,293]. Furthermore, they are also much more common in secondary, rather than in primary, glioblastoma [286,290,294,295]. In observations carried out by Balss et al. involving 14 children with glioblastoma, it was shown that the IDH1 mutation was present in only one case [290]. Convergent results were received by Hartmann et al. in a study that examined 1010 patients with diffuse gliomas. In the pediatric population, no IDH2 mutations were found, while IDH1 mutations were very rare in this patient group [296]. In a more detailed analysis of 43 patients with high-grade gliomas, Pollack et al. indicated that the IDH1 mutation does not occur in children under 14 years of age. In older children the mutation was reported in 35% of cases [297]. This is consistent with the results of other authors and emphasizes the importance of IDH status as a distinctive feature of high-grade gliomas in adults and children [285,286]. The miRNA assay may be helpful in identifying IDH. Wang et al. determined 23 miRNAs, whose presence determined the IDH1 mutation in glioblastoma cells. Moreover, they showed that they also determine the course of the disease. However, it should be noted that these observations were carried out in the adult population [298].

### 6.4. Epigenetic Nature of Glioblastoma

Sturm et al. proposed the division of glioblastomas into six subgroups due to their epigenetic nature. They also emphasized the IDH mutation status as a characteristic of GBM in the pediatric population. Moreover, they noted the mutually exclusive relationship between the IDH1 and H3F3A mutations [293]. H3F3A is a gene encoding H3.3 histones, which are nuclear proteins with an important role in the regulation of transcription and replication [299]. Many studies have demonstrated that mutations in this gene are often found in the case of glioblastomas in the pediatric population [300,301]. Wu et al., in their study, observed the prevalence of H3.3 alterations in 50 samples of pediatric Diffuse Intrinsic Pontine Gliomas (DIPGs) and 36 non-brainstem pediatric glioblastomas. The results showed that the H3F3A mutation affected 78% of DIPGs samples and 22% of glioblastoma [302]. Observations carried out by Schwartzentruber et al. on 48 samples of pediatric glioblastoma showed even greater prevalence of this type of lesion, with it affecting 31% of the samples [303]. The absence of the IDH1 mutation and the relatively high prevalence of the H3F3A mutation (21.4%) in pediatric high-grade glioma has also been confirmed in the work of Jha et al. Moreover, the authors noted the association of the H3F3A mutation with changes in miRNA levels among those patients. They identified 62 examples of those particles, which tissue titers were increased in the presence of mutations, and 35 which were low. Higher-level particles included miR-15a, miR-30e, miR-178c and miR-424 and concerned the K27M mutation. miRNAs, which titers depend on the status of the H3F3A mutation, were associated with the regulation of expression of genes responsible for cell proliferation and apoptosis. This group included FOXO1, FOXG1, CDK6 and MEIS. The authors also suggested that the gene expression profile with the K27M H3F3A mutation may be induced by the presence of miRNA [240]. An accurate understanding of this mechanism requires further research.

Sturm et al., in their work, also drew attention to the association of the H3.3 mutation with the TP53 mutation [272]. In the adult population, this type of mutation is less common, while among children with high grade gliomas, its prevalence is around 64% [240,304]. In their work, Jha et al. analyzed levels of miRNA depending on the mutation status of the TP53 gene. They showed that 63 miRNAs are specific for TP53-mutant cells. Overexpression affected 35 of them, while 28 were lowered [240]. These results were consistent with the analysis carried out by Ganci et al. for head and neck squamous cell carcinoma patients [305].

Studies on the pediatric population have shown a number of other pathways that are influenced by miRNA regulation. In addition to the axes mentioned above, Jha et al. noted the contribution of these particles to the control of the SMAD2/3 and calcineurin signaling pathways [240]. Some authors also noticed differences in the expression of individual genes, which are probably the targets of miRNA activity. This situation applies to RAF1, BDNF, SATB1, CDK6, and GABRA1, which have an important function in the pathogenesis of glioblastoma [87,284,306,307,308]. Similar observations were noted by Liang et al. in their study regarding the levels of miR-137 and miR-6500-3p in pediatric high-grade glioma cell lines. The authors noted that these particles exhibit anti-tumor activity by negatively affecting the expression of the KIF14, NCAPG and CENPE genes [174]. Earlier observations pointed out the role of KIF14 in promoting the development of other cancers such as breast, ovarian, lung as well as gliomas in the adult population [309,310,311,312]. Disturbances in NCAPG expression have been reported in leukemia and melanoma [313]. In addition, the role of the CENEPE gene was suggested in the pathogenesis of hepatocellular carcinoma (HCC) and breast cancer [314,315]. Giunti et al., in their work, indicated that the target of the aforementioned miRNAs were also genes that affect the pathogenesis of glioblastoma. The authors included GRIA1, SORL1, NUCKS1, SOX11, SAP30L, HTT, PXMP4, THRB, PSD3, SPN, AGPAT4, USP31, GRIK3, POM121L8P, TNRC6B, SNX29, HIPK2 and RIMKLA [172]. This shows the complexity of the relationship that governs the pathophysiology of the cancer process, and the special and multi-stage role that miRNA particles play. A thorough knowledge of different relationships will require a series of further studies.

## 7. Conclusions

Despite the continuous development of modern medicine, high-grade glioma still remains a serious problem, taking the lives of many patients every year. This is due to the complexity of their pathophysiology affecting the poor results of standard treatment. The research conducted in recent years on this group of tumors has shown the important role of miRNA in the course of gliomagenesis; however, lots of the miRNAs mentioned in this review are not specific to glioblastoma and are described in different pathologies.

These particles seem to participate in many stages of the development of the cancer process, such as proliferation, angiogenesis and the regulation of apoptosis. A thorough knowledge of the nature of miRNAs may be helpful in finding new, more effective forms of treatment and improving existing ones. This also applies to the pediatric population, in which much less is known about the nature of high-grade glioma in comparison to adult patients. A full understanding of the regulatory pathways of tumor cell metabolism may enable the development of new and more effective treatments for children with high grade gliomas, including miRNAs.

## Figures and Tables

**Table 1:** Presentation of microRNA (miRNA) molecules, divided into major groups of action: carcinogenesis modulants (apoptosis, invasiveness, angiogenesis, proliferation), tumor indicators (biomarkers), tumor development controllers (histological progression) and treatment effectiveness markers (treatment response). Arrows indicate = whether the levels of miRNA are increased, decreased or both.

Apoptosis (Tissue Level)	Invasiveness (Tissue Level)	Angiogenesis (Tissue Level)	Proliferation (Tissue Level)	Biomarkers (Tissue Level)	Histological Progression (Tissue Level)	Treatment Response (Tissue Level)
miR-10b ↓ miR-21 ↓ miR-34a ↑ miR-124 ↓ miR-182 ↑ miR-211 ↑ miR-221 ↑ miR-222 ↑ miR-326 ↓ miR-330 ↓ miR-335 ↓	miR-7 ↑ miR-10b ↑ miR-21 ↑ miR-29 ↑ miR-34a ↑ miR-107 ↑ miR-142 ↑ miR-146 ↓ miR-146b-5p ↑ miR-181b ↓ miR-181c ↓ miR-200 ↑ miR-203 ↓ miR-204 ↑ miR-655 ↓	miR-7-5p ↓ miR-17-5p ↓ miR-21 ↑ miR-29a ↓ miR-93 ↑ miR-125b ↓ miR-128 ↓ miR-155 ↓ miR-186 ↓ miR-210-3p ↑ miR-296 ↑ miR-429 ↓ miR-675-5p ↓	miR-7 ↑↓ miR-19a ↑↓ miR-21 ↑ miR-23 ↑↓ miR-29 ↑↓ miR-34 ↑↓ miR-101 ↑↓ miR-107 ↑↓ miR-122 ↑↓ miR-124 ↓ miR-128 ↓ miR-137 ↓ miR-140 ↑↓ miR-144 ↑↓ miR-145 ↑↓ miR-152 ↑↓ miR-155 ↑↓ miR-181 ↑↓ miR-181c ↑↓ miR-182 ↑↓ miR-186 ↑↓ miR-203 ↑↓ miR-204 ↑↓ miR-221 ↑↓ miR-222 ↑↓ miR-326 ↑↓ miR-384 ↑↓ miR-6500-3p ↑↓	miR-17 ↑ miR-19a ↑ miR-19b ↑ miR-21 ↑ miR-25 ↑ miR-34a ↓ miR-92b ↑ miR-106b ↑ miR-125b ↑ miR-128a ↓ miR-130a ↑ miR-132 ↓ miR-155 ↑ miR-181a ↓ miR-181b ↓ miR-181c ↓ miR-182 ↑ miR-198 ↓ miR-219-5p ↓ miR-221 ↑ miR-329 ↓ miR-335 ↑ miR-338-3p ↓ miR-483-5p ↓	miR-9 ↑ miR-15a ↑ miR-16 ↑ miR-17 ↑ miR-19a ↑ miR-20a ↑ miR-21 ↑ miR-25 ↑ miR-28 ↑ miR-130b ↑ miR-140 ↑ miR-184 ↑ miR-210 ↑ miR-328 ↑	miR-17-5p ↑↓ miR-21 ↑ miR-19a ↑↓ miR-19b ↑↓ miR-100 ↑ miR-101 ↓ miR-139 ↑↓ miR-143 ↑ miR-155 ↑ miR-181a ↓ miR-182 ↑↓ miR-193a5p ↑↓ miR-200a ↑↓ miR-200q ↑↓ miR-203 ↑↓ miR-204 ↑↓ miR-221 ↑↓ miR-222 ↑↓ miR-328 ↑↓ miR-490 ↑↓ miR-603 ↑↓ miR-873 ↑↓

**Table 2:** Expression of particular miRNA molecules detected in pediatric high-grade glioma (HGG) population.

Overexpression	Reduced Expression
miR-15a miR-17 miR-18a miR-19a miR-19b miR-20 miR-27a miR-92 miR-100 miR-106a miR-195 miR-497	miR-7-5p miR-124-5p miR-129-2-3p miR-137 miR-138-2-3p miR-139-3p miR-203a miR-218-5p miR-329-3p miR-770-5p

**Table 3:** Expression of particular miRNA molecules detected in adult HGG population.

Overexpression	Reduced Expression
miR-10b miR-21 miR-25 miR-26a miR-221 miR-222 miR-335 miR-451 miR-486 miR-501-3p	miR-7 miR-106a miR-124 miR-128 miR-129 miR-137 miR-139 miR-181a miR-181b miR-218 miR-323 miR-328

**Table 4:** Expression of particular miRNA molecules detected in pediatric HGG population compared with pediatric HGG population.

Overexpression	Reduced Expression
miR-17-5p	miR-137
miR-561-5p	miR-6500-3p
